# Green Chemistry Within the Circular Bioeconomy to Harness Chestnut Burr Extract’s Synergistic Antimicrobial Activity Against *Helicobacter pylori*

**DOI:** 10.3390/molecules30020324

**Published:** 2025-01-15

**Authors:** Maria Lucia Schiavone, Roberta Barletta, Alfonso Trezza, Michela Geminiani, Lia Millucci, Natale Figura, Annalisa Santucci

**Affiliations:** 1 Department of Biotechnology, Chemistry and Pharmacy, University of Siena, Via Aldo Moro, 53100 Siena, Italy; marialucia.schiavone@humanitasresearch.it (M.L.S.); r.barletta@student.unisi.it (R.B.); geminiani2@unisi.it (M.G.); lia.millucci@gmail.com (L.M.); 2 SienabioACTIVE, Università di Siena, Via Aldo Moro, 53100 Siena, Italy; 3 Department of Medical, Surgical and Neurological Sciences, University of Siena and Policlinico S. Maria alle Scotte, 53100 Siena, Italy; natale.figura@unisi.it; 4 ARTES 4.0, Viale Rinaldo Piaggio, 34, 56025 Pontedera, Italy

**Keywords:** chestnut burrs, circular bioeconomy, MBC, antimicrobial activity, *Helicobacter pylori*

## Abstract

Green chemistry principles are pivotal in driving sustainable and innovative solutions to global health challenges. This study explores a hydroalcoholic extract from *Castanea sativa* (chestnut) burrs, an underutilized natural resource, as a potent source of antimicrobial compounds against *Helicobacter pylori* (*H. pylori*). The extract demonstrated significant bactericidal activity, synergizing effectively with clarithromycin and showing additive effects with metronidazole. Remarkably, combining the extract with clarithromycin and sub-inhibitory concentrations of pantoprazole reduced clarithromycin’s Minimum Bactericidal Concentration (MBC) to just 1.56% of its original value. Mechanistic studies suggest that the extract’s polyphenolic compounds compromise bacterial membrane integrity, enhancing antibiotic uptake, while pantoprazole disrupts bacterial ATPase activity. This research highlights the critical role of natural product extraction within the framework of green chemistry, offering a sustainable and environmentally friendly alternative to synthetic antimicrobials. By harnessing bioactive compounds from plant sources, this approach addresses the pressing issue of antibiotic resistance while promoting the responsible use of natural resources. The findings underscore the transformative potential of green chemistry in developing effective, eco-conscious antimicrobial therapies that align with global sustainability goals.

## 1. Introduction

*Helicobacter pylori* (*H. pylori*) is a Gram-negative microorganism that colonizes the human stomach. It infects more than 50% of the whole population at the level of gastroduodenal mucosa, and the infection can lead to peptic ulcers and gastric cancer [1]. The current anti-*H. pylori* therapy includes a combination of a proton pump inhibitor (PPI), such as pantoprazole, with two or more of the following antibiotics: clarithromycin, amoxicillin, nitroimidazoles, quinolones and tetracyclines, administered for one or two weeks [2]. This treatment, however, is associated with side effects, occasionally fails to eradicate the infection (in 10–40% of patients) and is prone to selecting resistant strains at a notably high rate [3].

As of 2024, resistance rates increased to approximately 30% for clarithromycin, 61% for metronidazole, 35% for levofloxacin, and 6% for amoxicillin. In some areas, the resistance to clarithromycin can reach up to 96%, and metronidazole resistance can range from 1% to 100% [4].

This rising frequency of antibiotic resistance yields a reduction in the eradication rate of *H. pylori* infections. The high infection rate in low-income countries, combined with poor treatment compliance, underscores the urgent need for novel anti—*H. pylori* agents that exhibit rapid antimicrobial activity with minimal host toxicity.

Medicinal plants can offer a solution due to their wide range of biological properties displayed by their bioactive compounds. Thus, several naturally occurring substances with antimicrobial properties were investigated as potential alternatives to antibiotics, were or associated with antibiotics for the treatment of *H. pylori* infection, such as flavonoids, berberine, catechin, cinnamaldehyde, and ellagic acid [5]. However, in most cases, natural antimicrobials are limited by low potency, high cost, and by the toxicity of the extracted bioactive compounds.

Natural antimicrobials offer a promising alternative to conventional antibiotics due to their diverse bioactive properties and potential to mitigate antibiotic resistance. However, their broader application is often hindered by significant challenges. These include their inherently low potency, which necessitates higher doses to achieve the desired therapeutic effect; their relatively high costs of extraction and purification, which can be a barrier to large-scale use; and, in some cases, the toxicity of the extracted bioactive compounds when administered at effective concentrations. For example, many plant-derived polyphenols and flavonoids exhibit excellent antimicrobial properties, but their clinical utility is constrained by these limitations [5,6].

To address these challenges, innovative strategies are required, such as optimizing extraction methods to enhance yield and reduce costs, employing green chemistry techniques to isolate potent compounds with minimal toxicity, and identifying synergistic combinations with existing antibiotics to boost efficacy [7,8].

Recent advances, such as the development of sustainable extraction processes and the use of nanoparticles for bioactive compound delivery, hold promise in overcoming these barriers [9].

Additionally, focusing on compounds with low cytotoxicity profiles and improving compound stability through encapsulation methods could further expand the clinical applicability of natural antimicrobials [10].

These efforts are crucial to fully harness the potential of natural compounds in combatting antibiotic resistance.

*C. sativa* Mill. produces shells as primary by-products during chestnut processing. Upon peeling, the inner skin is separated and can be utilized as industrial fuel [11]. Usually, these shells are managed as waste and incinerated, representing a cost for farmers. In the context of a circular economy, chestnut burrs could contribute to sustainable waste management through the exploitation of their various bioactive properties, ultimately aiming to reduce environmental and economic impacts [7,12,13,14,15]. Furthermore, the use of *C. sativa* Mill. was also considered for its antimicrobial activity against *Enterococcus faecalis* and foodborne pathogens [11].

For most plant extracts, antibacterial activity resides in their phenolic compound content, in particular flavonoids and hydrolysable tannins, which affect the growth *H. pylori* through the prevention of bacterial adhesion to human gastric mucocytes, the disintegration of the outer membrane, and the inhibition of Vacuolating cytotoxin A (VacA cytotoxin) activity, which is one of *H. pylori*’s virulence factors involved in the development of inflammation and ulceration of the gastroduodenal mucosa [16,17,18,19].

Recent research also investigated the mechanism of action of plant extracts against *H. pylori* urease, a key enzyme enabling *H. pylori* to survive in the stomach by neutralizing stomach acid.

For instance, one study evaluated the urease inhibition activities of medicinal plants like *Acacia nilotica* and *Calotropis procera*, showing significant urease inhibition [20]. Flavonoids were additionally investigated for their urease-inhibitory properties due to the presence of hydroxyl groups, which allow them to inhibit urease by binding to the nickel in the enzyme’s active site [20].

These findings suggest that certain plant extracts and their compounds could become potential alternatives or additions in treating *H. pylori* infections by targeting host adhesion, membrane assembly, and enzymatic activity.

Building on our previous research [21], this study evaluates the antimicrobial activity of a hydroalcoholic *C. sativa* chestnut burr extract against *H. pylori* strain 10K, exploring its potential within a circular bioeconomy concept. In our previous work [21], we systematically characterized this extract. The chemical composition of chestnut burr extract was analyzed using advanced chromatographic and mass spectrometric methodologies [21]. UV monitoring was performed at 210 and 254 nm, utilizing a C18 Luna column and a binary mobile phase consisting of water/formic acid (99.5/0.5, *v*/*v*) and acetonitrile. Gradient elution allowed efficient separation of the extract’s components [21]. Mass spectrometry detection employed nitrogen as the nebulizing and drying gas, with mass spectra acquired across a range of 100–1500 *m*/*z* in both positive and negative ionization modes [21].

The analysis enabled the identification of key phenolic compounds based on their UV absorption profiles, mass spectral data, and retention times, compared to reference standards [21].

The chromatographic analysis at 254 nm revealed a clear profile of the chestnut burr extract, successfully identifying five major compounds: gallic acid, quinic acid, protocatechuic acid, brevifolin carboxylic acid, and ellagic acid [21]. Most spectral information was obtained in negative ionization mode, reflecting the acidic nature of these molecules. Among the identified compounds, gallic acid and ellagic acid were determined to be the most abundant components in the extract (all details and the methodology adopted for the chemical profile of the extract can be retrieved in reference [21].

This detailed characterization underpins the biological potential of chestnut burr extract, highlighting its potential relevance as an adjunct to current antibiotic treatments. Notably, our prior research had already demonstrated the antioxidant properties of the extract, as well as its antimicrobial effect against *E. faecalis* [11].

To fully elucidate the antimicrobial potential of the *C. sativa* burr extract, it is important to consider evidence from prior research that highlights the antimicrobial activity of its key phenolic constituents. Gallic acid and ellagic acid, identified as major components of the extract, are well-documented for their antimicrobial properties. Gallic acid has been shown to inhibit bacterial growth by disrupting membrane integrity and inducing oxidative stress in microbial cells [1]. Similarly, ellagic acid demonstrates antimicrobial effects by interfering with essential bacterial enzymes and inhibiting quorum sensing, a critical factor for bacterial virulence [3]. These findings underscore the potential roles of these compounds in driving the extract’s antimicrobial effects and provide a basis for further correlation analysis to confirm their contributions to activity against *H. pylori*.

In this work, Minimal Bactericidal Concentration (MBC) assays were carried out on the extract, both crude and in association with four therapeutic antibiotics (clarithromycin, metronidazole, levofloxacin and ampicillin) to assess its possible interactions with such antibiotics. Additionally, the antibiotic synergistic effect was evaluated and confirmed when the extract was combined with clarithromycin. Instead, when coupled with metronidazole, *C. sativa* extract presented an additive effect. Eventually, the current anti-*H. pylori* treatment, consisting of clarithromycin and pantoprazole, was simulated in vitro by adding the extract.

In our in vitro MBC studies and clinical treatment simulation, it was demonstrated that *C. sativa* chestnut burr extract, integrated within a circular bioeconomy framework, holds great promise as a sustainable addition to current anti-*H. pylori* therapy by enhancing antibiotic efficacy.

## 2. Results

### 2.1. Anti-Helicobacter Efficacy of Crude Extract Used Alone and in Association with Antibiotics

The Anti-Helicobacter efficacy of antibiotics and extracts used alone and in association was investigated through MBC, whose results are reported in Table 1.

**Figure 1:**
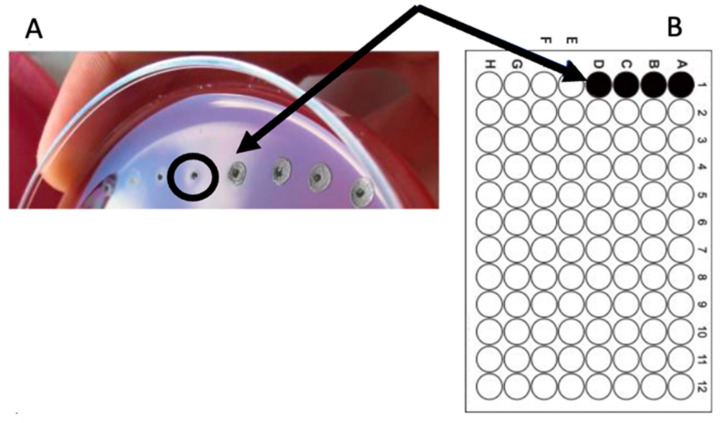
MBC determination of *C. sativa* shell extract alone. (**A**) Bacterial agar spot assay; (**B**) Corresponding microplate layout. Serial two-fold dilutions of *C. sativa* extract, at concentrations ranging from 25 mg/mL to 0.2 mg/mL, were carried out across the first column. The MBC value was 3.125 mg/mL (well E1, pointed to by a black arrow and circled in black).

The Minimum Bactericidal Concentration (MBC) of *C. sativa* chestnut burr extract was 3125 µg/mL, as shown in Figure 1. 

However, when combined with clarithromycin, the MBC of the extract significantly decreased to 195 µg/mL, indicating a potential synergistic effect between the two agents. Table 1 also illustrates that, in association with metronidazole, levofloxacin, and ampicillin, the MBC of the extract diminished by half, demonstrating an enhanced antibacterial effect in the presence of antibiotics.

The efficacy of the antibiotics, both with and without the hydroalcoholic *C. sativa* chestnut burr extract, was also investigated and highlighted in Table 1. Notably, the MBC values of all antibiotics, except ampicillin, were reduced when tested with the extract. Specifically, the MBC of levofloxacin decreased from 8 µg/mL to 4 µg/mL, the MBC of metronidazole from 128 µg/mL to 4 µg/mL, and the MBC of clarithromycin from 0.125 µg/mL to 0.025 µg/mL. Only the MBC of ampicillin remained unchanged in the presence of the extract, indicating no significant interaction between the two.

### 2.2. Synergistic Effect

Table 2 presents the Fractional Inhibitory Concentration Index (FICI) used to evaluate the putative synergistic effect for each extract–antibiotic combination.

**Figure 2:**
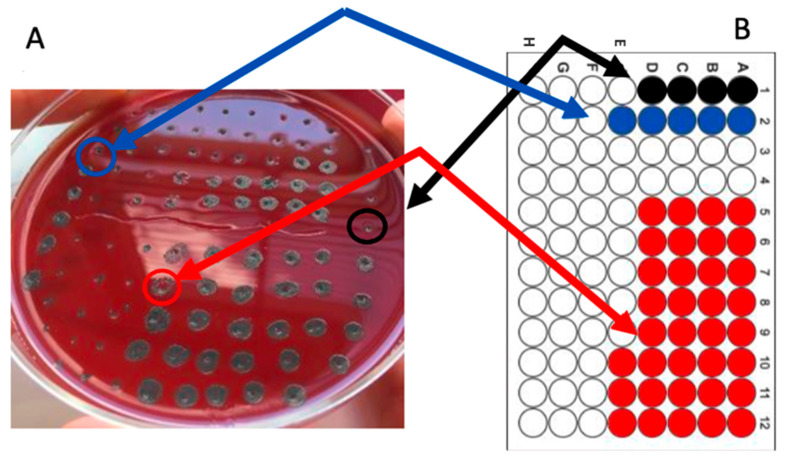
Determination of the Minimum Bactericidal Concentration (MBC) of *C. sativa* shell extract, clarithromycin, and their combination. (**A**) Bacterial agar spot assay; (**B**) Corresponding microplate layout. Colored wells indicate bacterial growth, whereas clear wells show no growth. The first and second column contain serial two-fold dilutions of the extract and clarithromycin alone, respectively, while the third and fourth columns represent negative controls. Wells A–H of columns 5–8 include both the extract and the antibiotic, with the extract diluted along the columns and the antibiotic diluted across the rows. Well E1, corresponding to the MBC value of the extract alone (3.125 mg/mL), is indicated by a black arrow on the agar plate and circled in black. Well F2 represents the MBC value of clarithromycin alone (0.25 mg/mL), indicated by a blue arrow on the plate and circled in blue, while well F9 denotes the MBC value of the combined extract/antibiotic, with its corresponding spot on the agar plate indicated by a red arrow and circled in red.

The association with clarithromycin (FIC index 0.309) yielded a synergistic behavior, as also illustrated in Figure 2; the association with metronidazole (FIC index 0.531) showed an additive effect, while no interaction was observed when the plant extract was associated with levofloxacin (FIC index 1.52) or ampicillin (FIC index 1.5).

### 2.3. In Vitro Simulation of the Clinical Anti-H. pylori Therapy

After assessing the synergistic effect between *C. sativa* and clarithromycin, an in vitro simulation of the triple therapy used in current clinical protocols for treating *H. pylori* infection was conducted. Pantoprazole, a proton pump inhibitor (PPI), was combined with clarithromycin. Additionally, *C. sativa* extract was introduced to pantoprazole (P), clarithromycin (CLA), and their combination (P + CLA), to evaluate its interaction with these agents (Table 3). 

**Figure 3:**
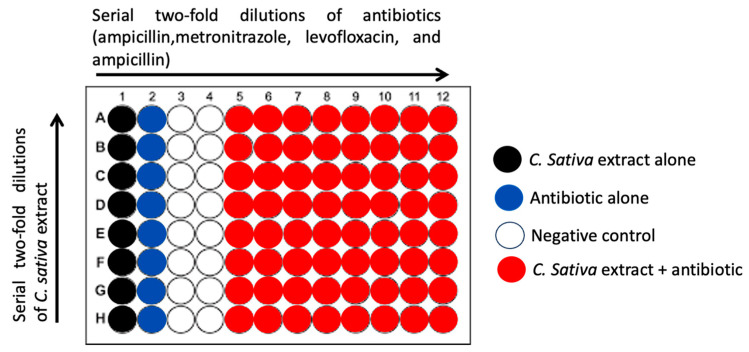
Synergistic effects through checkerboard analysis, showing a substantial reduction in the MBC of clarithromycin when combined with the extract, indicating improved antimicrobial efficacy. Determination of the synergistic effect between *C. sativa* extract and the antibiotic using the checkerboard method. The extract was serially diluted across the columns, while the antibiotic was diluted across the rows. Column 1 (black), 2 (blue), 5 to 8 (red) contain the extract alone, the antibiotic alone, and the extract and antibiotic combinations, respectively, while columns 3 and 4 represent negative controls.

No additive effect could be observed when the extract was combined with pantoprazole. However, Figure 3 shows that the MBC value significantly decreased from 195 to 48 ug/mL when the pantoprazole was added to the combination *C. sativa*/clarithromycin. Hence, there was a strong additive effect between clarithromycin and pantoprazole, suggesting potential improvements in treatment efficacy through this combination.

## 3. Discussion

Chronic gastritis, commonly caused by *H. pylori* infection, is one of the most prevalent chronic bacterial infections, second to dental caries. Conventional treatment typically involves two or more antibiotics combined with a proton pump inhibitor (PPI). However, the effectiveness of such a regimen has been diminishing annually. This is largely due to the development of acquired antibiotic resistance against *H. pylori*, given the bacterium’s high mutation rate. Consequently, interest in non-antibiotic substances with antibacterial properties is rising.

Plants serve as valuable alternatives for producing bioactive compounds with medicinal properties, and for centuries, traditional medicines, such as those in Chinese practices, utilized natural compounds to influence key biological processes, including cell growth, angiogenesis, survival, and programmed cell death, with many showing antioxidant, anti-inflammatory, and antimicrobial effects. The current literature body has demonstrated anti-*H. pylori* activity from traditional medicinal plants, such as *Rubus ulmifolius*, *Acacia nilotica* and *Calotropis procera* [20].

Furthermore, the observed synergy between the extract and antibiotics, particularly clarithromycin, may result from the ability of these phenolics to compromise bacterial defenses, enhancing antibiotic uptake. This hypothesis aligns with evidence that ellagic acid can inhibit efflux pumps, while gallic acid destabilizes the bacterial outer membrane, facilitating drug penetration [22,23]. A quantitative correlation study using purified compounds and bioassays would strengthen the understanding of their specific contributions.

The present work aimed to assess the anti-*H. pylori* effect of a *C. sativa* chestnut burr extract following a circular bioeconomy approach. In this approach, agricultural and industrial wastes are investigated for biological purposes, and by-products are reshaped into added-value resources, with the additional advantage of lessening environmental impacts [24]. For instance, coffee husks contain phenolic compounds with antioxidant properties, whilst pomegranate seeds can be employed as natural dyes for textiles [25,26].

Chestnut burrs are indeed considered agricultural waste, as they must be removed from the woods to allow chestnut tree growth, usually through burning. However, they were found to be rich in metabolites with antioxidant and anticarcinogenic properties, drawing interest in their reutilization for biological purposes [11].

In our previous work [21], we conducted a comprehensive characterization of the chestnut burr extract, employing advanced chromatographic and mass spectrometric techniques to elucidate its chemical profile. Utilizing UV monitoring at 210 and 254 nm with a C18 Luna column and a binary mobile phase comprising water/formic acid (99.5/0.5, *v*/*v*) and acetonitrile, the extract components were efficiently separated through gradient elution. This methodological approach ensured high sensitivity and specificity in identifying the bioactive compounds within the extract.

Mass spectrometry detection further augmented analytical precision, with nitrogen being utilized as the nebulizing and drying gas. Mass spectra were recorded over a broad range of 100–1500 *m*/*z* in both positive and negative ionization modes, enabling the detection of compounds with diverse chemical characteristics. The negative ionization mode proved particularly informative due to the acidic nature of many of the extract’s constituents.

The analysis revealed a clear chromatographic profile, particularly evident at 254 nm, identifying five major phenolic compounds: gallic acid, quinic acid, protocatechuic acid, brevifolin carboxylic acid, and ellagic acid. The identification was achieved through a combination of UV absorption profiles, mass spectral data, and retention times, corroborated by reference standards. Notably, gallic acid and ellagic acid emerged as the most abundant constituents, underscoring their potential role in the extract’s bioactivity.

This detailed chemical characterization provides a foundational understanding of the extract’s bioactive potential, particularly in the context of its antimicrobial properties. The predominance of gallic acid and ellagic acid is of significant interest, as such compounds are known for their antioxidant properties, which has been confirmed in previous research, along with their antimicrobial efficacy against *Enterococcus faecalis* [11].

The antimicrobial efficacy of the *C. sativa* chestnut burr extract can likely be attributed to the bioactivity of its phenolic compounds, particularly gallic acid and ellagic acid. Prior studies have demonstrated that gallic acid exerts its effects by disrupting bacterial cell membranes, leading to the leakage of intracellular components and eventual cell death [2]. Additionally, it has been shown to generate reactive oxygen species, creating oxidative stress that compromises bacterial survival. Ellagic acid, on the other hand, has been reported to inhibit enzymes critical for bacterial metabolism, such as urease in *H. pylori* [10]. It also impedes quorum sensing, reducing bacterial virulence and biofilm formation [6]. These mechanisms, supported by empirical data, strongly suggest that gallic acid and ellagic acid are central to the antimicrobial activity observed in this extract.

These findings strongly support the extract’s relevance as a promising adjunct to conventional antibiotic regimens, particularly in combating antibiotic-resistant pathogens such as *H. pylori*. Moreover, this robust characterization enhances the reproducibility of future studies, ensuring consistency in evaluating the extract’s therapeutic potential. At the same time, this study investigated the same extract, alone and in combination with common therapeutic antibiotics, on *H. pylori* strain 10K.

The Minimum Bactericidal Concentration (MBC) values proved that the extract had built-in antibacterial activity with an MBC value of 3125 µg/mL. However, this number decreased when the extract was combined with antibiotics, indicating potential interactions. Indeed, when in combination with clarithromycin, the chestnut burr extract showed a remarkable decrease in the MBC to 195 µg/mL, suggesting a synergistic effect. The combination with metronidazole, levofloxacin, and ampicillin resulted in a 50% reduction in the extract’s MBC value, indicating enhanced antibacterial effects. These data demonstrated that *C. sativa* extract enhances the efficacy of certain antibiotics, namely clarithromycin and metronidazole, against *H. pylori.*

The presence of the chestnut burr extract also affected the MBCs of the antibiotics. The most interesting decrement occurred with clarithromycin, as the MBC of clarithromycin alone was 0.125 µg/mL, but, in combination with the chestnut burr extract, it dropped significantly to 0.025 µg/mL, indicating a synergistic interaction. The MBC for metronidazole considerably decreased from 128 µg/mL to 4 µg/mL, and levofloxacin’s MBC was halved from 8 µg/mL to 4 µg/mL. No noteworthy change was observed in the MBC of ampicillin, implying no interaction with the extract.

To deeply investigate the interplay between the extract and antibiotics, the Fractional Inhibitory Concentration Index (FICI) was determined. The FICI values confirmed the observed synergistic effect with clarithromycin (FICI = 0.309) and an additive effect with metronidazole (FICI = 0.531). However, no interaction occurred in the presence of levofloxacin (FICI = 1.52) and ampicillin (FICI = 1.5), suggesting this extract as a potential agent to reduce the required doses of the administered antibiotics and mitigate antibiotic resistance and side effects. This acquires special significance for clarithromycin, whose MBC breakpoint for susceptibility is 0.25 µg/mL, while resistance is indicated by levels greater than 0.5 µg/mL [27].

The in vitro simulation of the clinical therapy combined pantoprazole (a proton pump inhibitor), clarithromycin (a standard antibiotic for *H. pylori* treatment), and the *C. sativa* chestnut burr extract to the extract/clarithromycin mix to evaluate their combined antimicrobial efficacy against *H. pylori*. The rationale for this combination lies in the distinct but complementary mechanisms of action of these agents. Pantoprazole inhibits bacterial ATPase activity and reduces gastric acidity, creating an environment less conducive to bacterial survival. Clarithromycin targets bacterial protein synthesis by binding to the 50S ribosomal subunit, while the chestnut burr extract, rich in phenolic compounds, disrupts bacterial membrane integrity and enhances antibiotic uptake.

The results showed a significant reduction in the Minimum Bactericidal Concentration (MBC) of clarithromycin in the presence of the chestnut burr extract and pantoprazole, dropping from 195 µg/mL to 48 µg/mL. This additive effect suggests a potentiation of clarithromycin’s antimicrobial activity by the extract when used in conjunction with pantoprazole. The observed reduction corresponds to approximately 75% of the MBC value for clarithromycin alone, highlighting the synergistic potential of this combination. These findings support the hypothesis that incorporating natural extracts into standard triple therapy could enhance treatment efficacy while potentially lowering the required antibiotic doses, thereby mitigating side effects and resistance risks.

Although the chestnut burr extract mechanism of action is not yet elucidated, the polyphenols are likely to target the outer membrane of Gram-negative bacteria and the cell walls of both Gram-negative and Gram-positive bacteria. Their interaction with phospholipids in the lipid bilayer and membrane proteins potentially disrupts membrane integrity, increasing its permeability, facilitating the antibiotic uptake, inhibiting the respiratory chain, and altering ion transport [28]. Moreover, PPIs like pantoprazole may inhibit bacterial ATPases, which maintain proton gradients needed for ATP production and cell survival. This inhibition could affect the bacterium’s ability to resist environmental stress, making it more susceptible to antibiotics and the action of plant extracts [29].

The additive effect observed when *C. sativa* chestnut burr extract was combined with metronidazole is particularly noteworthy, as the *H. pylori* 10K-tested strain showed moderate resistance to metronidazole with an MBC of 128 µg/mL, as the resistance breakpoint is >8 µg/mL [27]. However, when combined with the chestnut burr extract, the MBC of metronidazole decreased 32-fold to 4 µg/mL, below the threshold for sensitivity. This shift may be associated with the downregulation of the *hefA* gene, coding for an efflux pump involved in metronidazole resistance by taking the antibiotic out of the bacterial cell. The observed reduction in MBC when combined with *C. sativa* extract could be due to the disruption of this efflux system, probably through downregulation of *hefA* expression or inhibition of pump activity [30]. The extract’s polyphenols likely disrupt bacterial membrane integrity, increasing permeability, inhibiting respiratory chains, and enhancing antibiotic uptake, while PPIs like pantoprazole inhibit bacterial ATPase activity, compounding these effects [31].

In contrast, the lack of synergy with ampicillin or levofloxacin may be down to these antibiotics’ reliance on porin channels to cross the bacterial outer membrane [32]. Since these channels are not affected by the action of plant extract, no synergistic effect could be observed.

Importantly, this study does not aim to propose the chestnut burr extract as a novel antibiotic for clinical use. Instead, it emphasizes the potential of a circular bioeconomy framework to identify and valorize bioactive compounds from underutilized agricultural by-products. The primary focus is to repurpose chestnut burrs, an abundant and often discarded waste product, into a valuable source of bioactive compounds with promising applications in antimicrobial therapies. By leveraging circular bioeconomy principles, this research addresses two pressing global challenges: mitigating the environmental impact of agricultural waste and exploring sustainable methods to combat antibiotic resistance. The findings of this study illustrate that the chestnut burr extract, while not a replacement for conventional antibiotics, has the potential to act as an adjunctive agent, enhancing the efficacy of existing antibiotics. This approach underscores the transformative role of circular bioeconomy principles in therapeutic innovation. Rather than directly identifying new antibiotics, the study demonstrates how sustainable resource utilization can uncover novel bio compounds with significant therapeutic applications. In this case, the extract’s ability to synergize with antibiotics highlights its relevance within a sustainable and environmentally conscious framework for addressing global health challenges. In conclusion, the goal of this research is to propose a model for the sustainable utilization of agricultural by-products, showcasing their potential to contribute to innovative solutions in healthcare, while aligning with global sustainability and bioeconomy objectives.

## 4. Materials and Methods

### 4.1. Preparation of C. sativa Burrs (CSB) Extract

*C. sativa* burr extract was obtained as previously described [11]. The spiny burrs of *C. sativa* (Mill.), certified under the PGI (Protected Geographical Indication) Castagna del Monte Amiata (Reg. CEE n. 2081/92), were collected from Tuscany, a remarkable chestnut-producing region in Italy. After deep cleaning of the burrs and air-drying at room temperature until a stable weight was reached. Grinding into a fine powder and storing in sealed, dark plastic bags at −80 °C was necessary to preserve the bioactive compounds and prevent degradation caused by light or higher temperatures. The extraction involved immersing 330 g of the powdered burrs in 1 L of water (this ratio was optimized to maximize the extraction efficiency of bioactive compounds without oversaturating the solvent) and treating the mixture with 20 kHz ultrasonic waves for three hours at room temperature. The obtained aqueous extract, namely CSB, was then freeze-dried with a lyophiliser (Lyovapor L-200, Bhuchi, India) and stored at −32 °C (to prevent microbial contamination and preserve its bioactive properties over time) for additional experiments.

### 4.2. Bacterial Strain and Growth Conditions

*H. pylori* strain 10K was isolated from gastric neoplastic tissue obtained from a patient with diffuse histological variant gastric carcinoma and characterized in our previous works [33,34].

Growth occurred at –80 °C in Wilkins-Chalgren broth (Oxoid) supplemented with 20% glycerol (Sigma-Aldrich, St. Louis, MO, USA.).

For the experimental procedures, *C. sativa* extract was dissolved in DMSO (Sigma-Aldrich) and subsequently diluted in Brucella broth (Sigma-Aldrich) supplemented with cefsulodin (5 µg/mL), trimethoprim (10 µg/mL), vancomycin (10 µg/mL), and amphotericin B (5 µg/mL) (Sigma-Aldrich) to prevent contaminations.

The extract was evaluated alone and with commonly used antibiotics in triple therapy for *H. pylori* eradication: amoxicillin, clarithromycin, metronidazole, and levofloxacin (Sigma-Aldrich). Additionally, the antimicrobial activity of pantoprazole (Sigma-Aldrich), a representative of the proton pump inhibitor (PPI) family, was also assessed.

### 4.3. Determination of Minimal Bactericidal Concentration (MBC) and Minimal Inhibitory Concentration (MIC) of C. sativa Extract

To evaluate *C. sativa* extract’s MBC against *H. pylori*, 100 µL of diluted *H. pylori* suspension (containing 10^8^ CFU/mL) were added to a 96-well microplate (Sarstedt) in 100 µL of Brucella broth with FBS (Sigma-Aldrich) containing serial two-fold dilutions of *C. sativa* extract, at concentrations ranging from 25 mg/mL to 0.2 mg/mL. Plates were incubated in a microaerobic atmosphere at 37 °C. After 24 h incubation, two μL of mixture from each well were subcultured onto Brucella agar plates with FBS, which were then incubated in a microaerobic environment at 37 °C for 3–5 days. Afterwards, the plates were inspected for the presence of colonies, and the lowest concentration of *C. sativa* chestnut burr extract in broth, whose subculture on agar showed a complete absence of growth, was considered the MBC.

The MBC value was also assessed for all four antibiotics, alone and combined with the extract, using the same experimental procedure.

Three replicates were conducted, and data were presented as an average.

### 4.4. Antibiotic Synergy Determination

To evaluate its prospective synergistic effect, *C. sativa* extract was tested in association with each antibiotic among those commonly used in triple therapy for *H. pylori*: amoxicillin, clarithromycin, metronidazole, and levofloxacin through the checkerboard dilution method, illustrated in Figure 3.

Using 96-well plates, the experiment was set up by carrying out a serial two-fold dilution of the extract (final volume 200 μL and the given antibiotic across the first and column, respectively. Brucella broth supplemented with 10% inactivated fetal calf serum (Sigma-Aldrich), cefsulodin (5 µg/mL), trimethoprim (10 µg/mL), vancomycin (10 µg/mL), and amphotericin B (5 µg/mL) to prevent contamination, was dispensed into the wells. Then, both the extract and the antbiotic were posed in the remaining columns and serially two-fold diluted across the columns and the rows, respectively.

2 µL of *H. pylori* suspension in Brucella broth (containing 10^8^ CFU/mL) was inoculated into the 96 wells, each containing a specific concentration of the chestnut extract and given antibiotic. The plates were incubated under microaerobic conditions at 37 °C for 24 h.

Then, 2 µL from each well were subcultured onto Columbia agar (Sigma-Aldrich) plates supplemented with 10% horse blood (Sigma-Aldrich), 10% inactivated fetal calf serum, cefsulodin (5 µg/mL), trimethoprim (10 µg/mL), vancomycin (10 µg/mL), and amphotericin B (5 µg/mL). The plates were incubated under microaerobic conditions at 37 °C for 3–5 days.

The Minimum Bactericidal Concentration (MBC) was the lowest concentration of either the extract or the antibiotic in the broth, fully inhibiting bacterial growth on the agar subculture. To ensure the accuracy and reliability of the results, the tests were performed in duplicate and repeated three times, and the data were shown as average.

The interaction between the chestnut extract and antibiotics was evaluated using the Fractional Inhibitory Concentration Index (FICI). This index was calculated using the formula: FIC1 + FIC2 = FICI, where FIC1 stands for the MBC of the antibiotic in combination with the extract divided by the MBC of the antibiotic alone, and FIC2 represents the MBC of the extract combined with the antibiotic divided by the MBC of the extract alone. The FICI values were then interpreted to assess the interaction type. A synergistic effect yields a FICI value ≤ 0.5, and an additive effect a FICI value > 0.5 to 1. In case of no interaction, FICI is in the range of >1 to ≤4, whereas when there is an antagonistic effect, the FICI is >4.

### 4.5. Simulation of the In Vivo Multitargeted Therapy

To simulate the in vivo treatment of *H. pylori*, the Minimum Bactericidal Concentration (MBC) of clarithromycin in combination with the *C. sativa* chestnut burr extract was evaluated in the presence of pantoprazole. This experiment aimed to mimic the conditions of triple therapy used in clinical settings. Pantoprazole, a proton pump inhibitor (PPI), was included in the assay at a concentration of 8 µg/mL, which is four-fold lower than its MBC, as determined in preliminary tests. Brucella broth was used as the medium, supplemented with the necessary additives to support *H. pylori* growth. The inclusion of sub-inhibitory levels of pantoprazole was intended to evaluate its potential role in enhancing the antimicrobial activity of clarithromycin and the extract by disrupting bacterial ATPase activity and altering proton gradients critical for *H. pylori* survival.

## 5. Conclusions

This study aligns with circular bioeconomy principles by investigating *C. sativa* chestnut burr extract, an agricultural by-product, for its bioactive potential against *H. pylori* strain 10K. In vitro assays demonstrated significant antibacterial activity, with the extract synergistically and additively enhancing clarithromycin and metronidazole efficacy, respectively. The reduction in Minimum Bactericidal Concentrations of these antibiotics underscores the extract’s potential to combat antibiotic resistance and lower conventional drug dosages.

The potentiation of clarithromycin activity by sub-inhibitory concentrations of pantoprazole further suggests that combining plant extracts with PPIs could enhance therapeutic outcomes due to the polyphenolic compounds within the extract. Gallic acid, for example, has been shown to disrupt bacterial cell membranes and inhibit biofilm formation, mechanisms that may contribute to the observed enhancement of antibiotic efficacy [3]. Ellagic acid has been reported to target bacterial virulence factors and interfere with quorum sensing, which could further potentiate the activity of antibiotics such as clarithromycin [4]. Although the study does not pinpoint a single active compound responsible for the extract’s effects, the observed reduction in the Minimum Bactericidal Concentration (MBC) of clarithromycin and metronidazole in combination with the extract strongly suggests a synergistic interaction. This synergy likely arises from the combined action of multiple phenolic compounds targeting different bacterial processes. For instance, the polyphenols in the extract may increase bacterial membrane permeability, facilitating the uptake of antibiotics and enhancing their intracellular activity [5,6].

The extract’s action parallels findings for other plant-derived products, such as pomegranate peel extract, which enhances antibiotic efficacy by increasing membrane permeability and inhibiting efflux pumps [7]. Though further studies are needed to elucidate the exact mechanisms, these results highlight the potential of natural compounds as adjuncts in antimicrobial therapy. Future research should focus on isolating and characterizing key bioactive molecules using advanced methodologies like metabolomics and bioinformatics.

In conclusion, the *C. sativa* chestnut burr extract represents a sustainable, green, and cost-effective antimicrobial agent capable of enhancing antibiotic efficacy while addressing resistance challenges. Although the identification of the specific responsible compounds remains a challenge, the extract’s demonstrated synergy with clarithromycin and metronidazole underscores its relevance in developing sustainable and effective antimicrobial strategies within a circular bioeconomy framework.

## 6. Patents

Santucci, A.; Figura, N; Millucci, L.; and Schiavione, M.L., (2020). *Hhydroalcoholic extract from chestnut burr for antibacterial usage* (Patent No. 102020000018592). Ministry of the Environment and Protection of Land and Sea.

## Figures and Tables

**Table 1:** Anti-*H. pylori* efficacy of antibiotics and crude extract used alone and associated with antibiotics. The MBC values are reported in μg/mL.

*C. sativa* Extract/Antibiotic	MBC μg/mL
*C. sativa*	3125
Clarithromycin	0.125
Metronitrazole	>128
Levofloxacin	8
Ampicillin	0.007
*C. sativa*/Clarithromycin	195/0.025
*C. sativa*/Metronitrazole	1625/4
*C. sativa*/Levofloxacin	1625/4
*C. sativa*/Ampicillin	1625/0.007

**Table 2:** Fic index calculation and type of activity of antibiotics used in association with *C. sativa* crude extract. (FICI) was calculated for each association by using the following formula: FIC1 + FIC2 = FICI, where FIC1 was the MBC of drug 1 in combination with the extract divided by the MIC of the same drug tested alone, and FIC2 was the MBC of drug 2 in combination divided by the MBC of drug 2 tested alone. The FICIs were interpreted as follows: synergistic effect, FICI ≤ 0.5; additive effect, FICI > 0.5 to ≤1; no interaction, FICI > 1 to ≤4; antagonistic effect, FICI > 4. The experiment was performed in triplicate; data are presented as mean.

Compound	FIC1	FIC2	FIC Index	Activity
Clarithromycin	0.061	0.248	0.309	Synergistic
Levofloxacin	0.52	1	1.52	No interaction
Metronidazole	0.5	0.031	0.531	Additive
Ampicillin	0.5	1	1.5	No interaction

**Table 3:** Variations in the MBC of the *C. sativa* extract when combined with pantoprazole (C + P), clarithromycin (C + CLA), and both pantoprazole and clarithromycin (C + P + CLA). In detail, in Table 3, the in vivo simulation of the treatment of *H. pylori* infection is reported, in which two antimicrobial substances are simultaneously used in the presence of a PPI. All tests were carried out using Brucella broth containing pantoprazole at a concentration four-fold lower than its MBC determined in preliminary tests, i.e., 8 μg/mL. The experiment was performed in triplicate; data are presented as mean values. C = *C. sativa* extract; P = pantoprazole; CLA = clarithromycin.

Combination	MBC [μg/mL]
C	3125
C + P	3125
C + CLA	195
C + P + CLA	48

## Data Availability

The original contributions presented in the study are included in the article. Further inquiries can be directed to the corresponding author/s.
